# Editorial: Altered expression of proteins in cancer: function and potential therapeutic targets, volume II

**DOI:** 10.3389/fonc.2023.1242855

**Published:** 2023-07-18

**Authors:** João Pessoa, Maria Teresa Valenti, Nadège Bellance, Paula Chiarella, Tamrat Abebe, Lorenzo Gerratana, Carlos Pérez-Plasencia, Julia Kzhyshkowska

**Affiliations:** ^1^ CNC - Center for Neuroscience and Cell Biology, University of Coimbra, Coimbra, Portugal; ^2^ CIBB - Center for Innovative Biomedicine and Biotechnology, University of Coimbra, Coimbra, Portugal; ^3^ Department of Neurosciences, Biomedicine and Movement Sciences, University of Verona, Verona, Italy; ^4^ INSERM U1211, Rare Diseases: Genetic and Metabolism, University of Bordeaux, Bordeaux, France; ^5^ Department of Experimental Oncology, Instituto de Medicina Experimental, Academia Nacional de Medicina de Buenos Aires, Ciudad Autónima de Buenos Aires, Argentina; ^6^ Department of Microbiology, Immunology and Parasitology, School of Medicine, Addis Ababa University, Addis Ababa, Ethiopia; ^7^ Department of Medical Oncology, CRO Aviano, National Cancer Institute, IRCCS, Aviano, Italy; ^8^ Laboratorio de Genómica, Instituto Nacional de Cancerología, Tlalpan, Mexico; ^9^ Laboratorio de Genómica Funcional, Unidad de Biomedicina, FES-IZTACALA, Universidad Nacional Autónoma de México, Tlalnepantla, Mexico; ^10^ Institute of Transfusion Medicine and Immunology, Mannheim Institute for Innate Immunosciences (MI3), Medical Faculty Mannheim, Heidelberg University, Mannheim, Germany; ^11^ German Red Cross Blood Service Baden-Württemberg – Hessen, Mannheim, Germany; ^12^ Laboratory for Translational Cellular and Molecular Biomedicine, Tomsk State University, Tomsk, Russia; ^13^ Laboratory for Gene Technology, Siberian State Medical University, Tomsk, Russia

**Keywords:** cancer, protein expression levels, up-regulation, down-regulation, therapeutic target

## Introduction

Cancer cells and cells of the tumor microenvironment (TME) show extensive biochemical alterations, which provide multiple opportunities for developing innovative strategies for diagnosis, therapy, and prognosis. The disrupted metabolism of cancer cells (1) and immune cells of the TME (2) is controlled by several protein families. As such, it is not surprising that many of them have their cellular levels significantly increased or decreased. In the first volume of the Research Topic “*Altered expression of proteins in cancer: function and potential therapeutic targets*”, we provided an update on proteins whose cellular levels are altered in cancer, highlighting their promise for diagnosis and therapy. The second volume of this Research Topic complements the first one. It contains 9 original research articles and 5 review articles. In the following sections, we summarize the main concepts and findings of these studies, grouped according to the main physiological or pathological role of each protein.

## Gene expression

Proteins with altered cellular levels in cancer cells include those regulating gene expression. In prostate cancer, Yang et al. investigated the interactions of the competitive endogenous RNA regulatory network associated with the forkhead box A1 transcription factor. Its up-regulation correlated with the down-regulation of the dual specificity phosphatase 2 (DUSP2). DUSP2 overexpression reduced cell proliferation and migration. Alterations in gene expression can cross-talk with environmental factors, including hypoxia (decreased oxygen availability) (3). Guo et al. reviewed the mechanistic interactions between the hypoxic response and the Notch signaling pathways, proposing a combinatorial therapeutic strategy targeting both pathways. Depending on the cancer type, the Notch pathway can be oncogenic or tumor-suppressive. Gene expression can also be influenced by nuclear pore proteins, the nucleoporins. Singh et al. demonstrated that the Nup88 and Nup62 nucleoporins were up-regulated in head and neck cancer samples and cell lines. Nup88 was stabilized by Nup62 and enhanced cell proliferation, through gene expression alterations mediated by the NF-κB transcription factor.

These studies exemplify the multiple direct and indirect players involved in gene expression and their complex regulation, whose disruption in cancer cells affects the levels of messenger RNA (mRNA) and translated proteins.

## Protein phosphorylation and degradation

Cellular levels of functional proteins are also determined by the equilibrium between their phosphorylation and degradation. These processes, also governed by proteins, can be disrupted in cancer cells. Pidkovka and Belkhiri reviewed the impact of the up-regulated transmembrane AXL receptor tyrosine kinase in gastrointestinal cancers and its potential role as a therapeutic target. Inhibition of this protein with small molecules or antibodies is currently being investigated in several clinical trials. In addition, Tang et al. reviewed the relevance of intracellular non-receptor protein tyrosine phosphatases across multiple cancer histologies. Some of these proteins seem to play a dual role in specific cancer types. They are also emerging as targets for immunotherapy and specific inhibitors have shown promising activity. Alterations in protein degradation levels are also found in cancer cells (4). Zhou et al. demonstrated the impact and suggested the therapeutic role of up-regulated MMP1 zinc-dependent endopeptidase in the progression and dedifferentiation of papillary thyroid cancer into poorly differentiated or anaplastic thyroid cancer. Another essential proteolytic mechanism disrupted in cancer involves ubiquitin ligation, which targets proteins for proteasomal degradation (5). Guo et al. reviewed the dual role of tripartite motif 31 (an E3 ubiquitin ligase) in different cancer types. Its oncogenic or tumor-suppressive roles (depending on the histology) could result from differential cellular levels in its isoforms that could be involved in different functions. In addition, Jin et al. demonstrated that the ubiquitin-specific peptidase 20 (a deubiquitinating enzyme) was down-regulated in colorectal cancer. Nevertheless, inducing its overexpression in representative cell lines increased cell migration and invasion, suggesting a potential therapeutic strategy.

These studies confirm that proteostasis is deregulated in cancer cells. The same regulatory protein can be oncogenic or tumor-suppressive across cancer types, underlining the complexity of these regulatory mechanisms.

## Cell division and apoptosis

Altered protein levels can change cell homeostasis through several processes, including cell division and programmed cell death. One of the critical stages in cell division is chromosome segregation during the anaphase stage of mitosis, in which sister chromatids are attached to microtubules *via* kinetochores (6). Leng at al. investigated the mechanism causing up-regulation of the NDC80 kinetochore complex component in epithelial ovarian cancer. Its knockdown decreased proliferation, invasion, and migration in cell lines and decreased tumor growth in mice. Together with cell division blockade, apoptosis induction is, despite its limitations, a promising emerging anticancer strategy (7). Delgado-Waldo et al. used a combination of three small molecules for inducing apoptosis in three cervical cancer-derived cell lines. The approach involved the simultaneous inhibition of complex I of the mitochondrial respiratory chain, lactate dehydrogenase A, and DNA topoisomerase II, affecting multiple metabolic pathways. In addition, Pandey et al. characterized the non-apoptotic function of SMAC/diablo in lung cancer. Although this mitochondrial protein is generally pro-apoptotic, its knockout in lung cancer cells activated apoptosis and inhibited proliferation and migration. It also decreased tumor growth in mice.

These studies introduce novel strategies to interfere with cell division or to induce apoptosis, which provide promising outcomes against uncontrolled proliferation, the most distinctive feature of cancer cells.

## Tumor microenvironment, angiogenesis, and metastasis

The effects of altered protein levels can reach beyond their cell of origin. Through proteomics, Akhtar et al. identified differentially expressed proteins in early-stage gallbladder cancer. These proteins were mostly associated with neutrophil degranulation and extracellular matrix remodeling, which might promote cell invasion. Alterations in the TME are correlated with enhanced angiogenesis. In the major types of solid tumors, most noncancerous cells are tumor-associated macrophages (TAMs), which control both cancer cell proliferation and tumor angiogenesis (8, 9). In our Research Topic, Kazakova et al. studied the impact, in colon and rectal cancer, of the pro-angiogenic S100A4 calcium-binding protein and the integrin-binding secreted phosphoprotein 1, as well as the anti-angiogenic SPARC calcium-binding protein, expressed by TAMs. Although their up-regulation indicated poor prognosis, neoadjuvant chemotherapy/chemoradiotherapy converted S100A4 into a more favorable prognosis marker. Moreover, Ali et al. reviewed the impact of the TME and exosomes in the formation of metastases in the brain. Despite their harmful impact, exosomes could be exploited as a liquid biopsy technology, for the non-invasive diagnosis of brain metastases.

These studies demonstrate that proteins related to the propagation of cancer features are promising tools for diagnosis and prognosis.

## Concluding remarks

**Figure 1:**
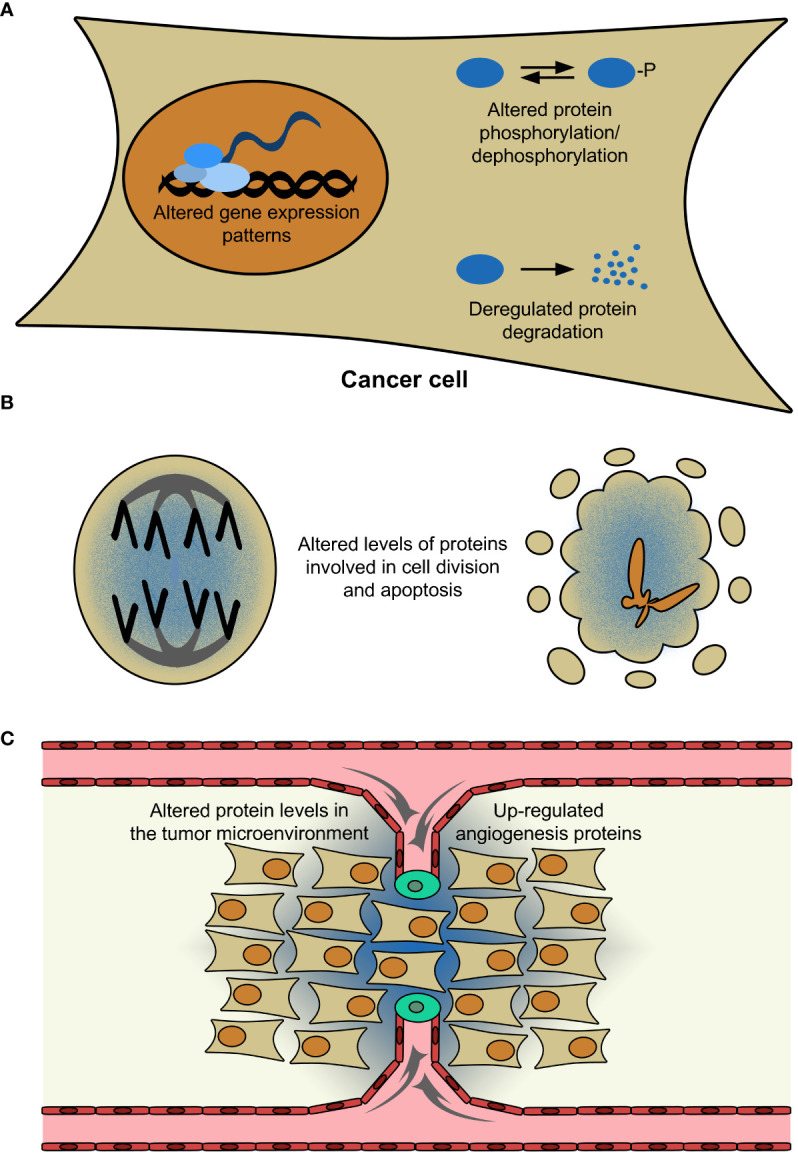
Causes and consequences of altered protein levels in cancer cells. **(A)** In the nucleus of a cancer cell (orange), up-/down-regulated transcription factors (light-blue) modify the levels of transcribed messenger RNA (dark-blue). The resulting proteins (blue) can be phosphorylated or degraded, in an equilibrium whose disruption affects their functional levels. **(B)** Altered protein levels can deregulate cell division and apoptosis (whose therapeutic induction up-regulates pro-apoptotic proteins). **(C)** They can also affect the tumor microenvironment and enhance angiogenesis (represented by gray arrows), which will promote cancer cell proliferation. For simplicity, only tumor (dark-yellow), endothelial (red), and tip (green) cells are represented. In panels **(B, C)**, protein up-regulation is represented as a blue gradient.

The data reported in the present Research Topic provide novel data about regulatory proteins expressed in cancer cells and in immune cells of the TME. These regulatory proteins can control not only primary tumor growth and metastasis but also the efficiency of anti-cancer therapies. These proteins include transcription factors, which affect the levels of transcribed mRNA and translated protein. They also include enzymes involved in protein phosphorylation, dephosphorylation, and degradation, whose altered expression deregulates the levels of functional proteins (**Figure 1A**). These alterations can affect processes including cell division and apoptosis (**Figure 1B**). Altered protein levels can exert effects on a larger scale, by modifying the TME and promoting tumor angiogenesis (**Figure 1C**). Since these alterations are responsible for cancer development and progression, they are also potential new molecular tools for its diagnosis and therapy.

## Author contributions

JP, MV, and JK wrote the article with input from all authors, who contributed insight and approved the final manuscript.
